# On the Source and Influence of Malaria in the South-west

**Published:** 1848-02

**Authors:** A. G. Lawton

**Affiliations:** Marshall, Missouri


6. *On the Source and Influence of Malaria in the South-west.* By Dr. A. G. LAWTON, of Marshall, Missouri.

It was the opinion of Labaraque, that the effluvia arising from decomposing animal matter had no deleterious influence on the human constitution; in support of which able proof is adduced. (Journal of Foreign Medicine for 1828, vol. ii., page 381, E. Littell, Phila.)

This also was the opinion of Bancroft, and some others. Whatever combination of circumstances might be necessary to produce this poison, one thing is certain, that is, that water is one of the elements always necessary to its production; and that the water must be in small quantities, is evident from the fact, that the earth sends forth this effluvia in the greatest abundance in the last stage of its drying. I do not believe that decomposing animal matter will always necessarily produce it, for it requires a union of elements brought together under certain circumstances, implying a certain degree of heat, moisture, and matter, aided by a slow grade of decomposition, in order to the perfect developement of this deleterious effluvia; hence Labaraque was right when he said as above quoted, he having reference to the *rapid* decomposition of dead bodies, for here we do not have the manner of decay, or the circumstances combined which are necessary to produce it.

What I conceive to be the most prolific source of malaria is, animal matter in minute fragments, mingled with vegetable matter in a process of *slow* decay. Where wash-water is being constantly thrown out, around houses, in by and shady places, amongst rubbish, where old bones and vegetable matter are left to rot by slow degrees, it cannot fail, after a long time, to give rise to, or produce a pestilential effluvia, especially in very dry seasons ; for it is generally the case that wash-water contains more or less animal matter, and that, too, of a kind favorable for the generation of miasmatic exhalations.

It will be found generally the case, that the sickly season does not commence until the thermometer falls a little from its extreme point, and the sky assumes that peculiar veiled appearance that it has in the latter part of the summer, and the autumnal months, or in very dry times. The fore part of the season is the healthiest part of the year, for the heavy rains and storms of the spring have swept all noxious matter from the air, and left it rightly and equally tempered. It generally happens, that as the drought increases the dews lessen, until the healthful moisture of the air is gone. Now, at this time, the intensity of the sun's rays is on the wane, for this occurs in the latter part of summer and fall, and the hottest days of the year are in the last of June and the fore part of July, and the most sickly time is in September, and sometimes in October.

When the dews begin to lessen a little, the sky assumes a dark or red appearance, and the sun's rays are a little blunted, as though its rays were in some way obstructed ; until now, the evaporation from the earth's surface is not very poisonous, but now the evaporation from all the high lands, and drier part of the country, is very trifling, and daily lessen ; and now the drought increases. At this time the water is mostly gone from the earth's surface ; the ground parched by drought ; the atmosphere already deprived of all healthy sources, whereby it might be supplied with moisture, at the very time when there is the greatest necessity for it ; and this lack of humidity in the air must be supplied from some source, and this vacancy is soon filled up by an increased and rapid evaporation, from the half-dried swamps, stagnant pools, sinks, gutters, sewers, and from the banks and bottoms of streams, where the water has fallen and left the mud exposed ; from these sources the moisture of the air is still maintained, and the atmosphere is still humid.

But now the equilibrium of the air is partially destroyed, it being over-dry in some places, and excessively humid in others ; for this humidity is not like that humidity which comes from clouds and storms of rain, which in itself is harmless, but it is a humidity formed of noxious vapors, constantly springing from decomposing matter; which, being either chemically united or mechanically mixed with some elementary principle evolved from decomposing matter, is thereby rendered much heavier than humidity from other sources, and becomes incapable of rising very high in the air, unless it is forced up by some fixed current of wind; and thus it happens that, under these circumstances, the atmosphere becomes unequally tempered. And now the moisture of the earth is so far exhausted on all the uplands, that it cannot afford much material for evaporation, and this process is of necessity limited to a small surface, that is, from swamps, marshes, ponds, streams, etc., from which places evaporation is very much increased, and the vapors rise in denser volumes, bringing up the poisonous exhalations from these places, where vegetation, flies and reptiles, have fallen and rotted for ages; where the matter, after being long steeped, is every year dried down, in the latter part of which process there is formed, and evolved from this mass, by the action or re-action of decomposing elements on themselves, a something which we call *malaria*, long known by its effect, being followed by a certain train of diseases peculiar to themselves, and known to be produced by no other morbific effect.

Now, under these circumstances, should the wind be low, which is generally the case, sometimes a dead calm prevails, or the wind sets lightly from an eastern direction, blowing with a current just strong enough to move the poisonous vapor from its resting place, and spread it over the country; and should this state of things exist long, and progress to an intense degree, a sickly time must inevitably follow; and when this state of things does progress to an intense degree, it is generally brought to bear most severely on the community in the autumnal months, increasing as the cold season approaches, or until some violent storm or frost occurs; when the reverse of this happens, the sickness of the season is very much modified, assuming less of an epidemic character.

I am convinced, from experiments and observations, that wood is capable of generating an immense amount of this poison; decomposition of the ligneous fibre is slow, and where it is long exposed to wet and dry, as in marshes, pools, and about houses, for many years, I believe it will produce a pestilential effluvia. If pools of water, standing in the blue clay on these prairies, have nothing of the wood kind in them, the weather being very dry and hot, so the water does not move in or out of these pools, in two or three months the water becomes perfectly sweet and clear, and if it is not agitated from the bottom, it may be drunk or used with impunity; but if these pools contain logs, chips, brush, leaves, or wood of any kind, that is, old and in a decaying state, then the water never becomes either sweet or clear, but assumes a dark color, and the drier the weather, the blacker it gets, when it becomes an active and certain poison, producing on the human constitution sudden and alarming effects, accompanied with excessive vomiting and purging, extreme prostration, and death. And how much sickness there is produced by drinking water impregnated with this poison, is difficult to say, as water holds it in solution in every degree, from the minutest quantity, which would require years to affect the constitution, up to a degree of concentration sufficient to destroy life in a few hours.

The Indians suffer less from these causes than the whites, and the reason is obvious; they seldom live long in a place, constantly moving from one place to another, and often burn their tents, and erect new ones, and a fire is kept constantly burning in the centre of the tent, around which they sleep; they do not live long enough in a place for the accumulation of filth to become an effectual source of disease.

Although these countries, as a general thing, are not subject to extreme atmospheric vicissitudes, yet it sometimes happens that we have inflammatory diseases in the cold half of the year, as inflammation of the lungs, pleura, and the like; and although they occur at a season of the year when the air is free from all noxious exhalations, yet they generally assume that grade and type which is common to malarial fevers, and they generally fall most intensely on those living nearest the focus of miasmal emanations; hence I count them as malarial; and taking this view of the subject, I have long since concluded to bleed less and give quinine more, and with this treatment I have been much more fortunate that when I used the lancet.

But antimony is our main reliance in these cases. Tart. antimony, judiciously administered, will seldom disappoint the physician's expectations. As soon as the pulse falls, and the expectoration becomes a little modified, I add quinine to the antimonial powders; and when the antimony is no longer indicated, I continue the quinine, combined with ipecac., and sometimes Dover's powder. (My Dover's powder is made with the nitrate of potassa in place of the sulphate.)

I have only one thing more to add at this time, and that is, with respect to the use of quinine, combined as described in my former paper. (N. Y. Jour. of Med. vol. 8, p. 69.) Make a powder of quinine, camphor and pulvis Doveri, then the powder will contain quinine, nitre, opium, ipecac., and camphor; now, if you increase the ipecacuanha a little, you will have a better powder for winter fevers, for ipecac. increases the effect of quinine very much, especially in fevers that verge towards the continued type. I look upon opium and camphor as important additions to quinine, unless contra-indicated, and we seldom meet with a case where nitre is not admissible. In treating fevers, there are many indications to be fulfilled, some of which quinine alone would not effectually meet. Quinine sometimes operates too locally, and the addition of camphor gives it a more general searching effect; and if the effect should not be increased, I believe it is more effectual by being more particularly directed to certain indications to be fulfilled, which is just what we should be led to expect from *a priori* reasoning; and in addition to all this, it sometimes becomes necessary to add a more potent and diffusible stimulant, as brandy.—*N. Y. Med. and Surg. Journal.*

